# Early and midterm outcomes of a bentall operation using an all-biological valved BioConduit™

**DOI:** 10.1186/s13019-022-02073-5

**Published:** 2022-12-19

**Authors:** Roxana Botea, Yoan Lavie-Badie, Alexandru Goicea, Jean Porterie, Bertrand Marcheix

**Affiliations:** 1 Department of Cardiovascular Surgery, Rangueil University Hospital, Toulouse, France; 2 Department of Cardiology, Rangueil University Hospital, 1, Avenue Jean Poulhès, TSA 50032, 31059 Toulouse Cedex, France; 3 Department of Cardiovascular Surgery, Nicolae Stancioiu Heart Institute, Cluj-Napoca, Romania

## Abstract

**Objectives:**

To analyze the midterm results of aortic root replacement using the valved, all biological, No React®, BioConduit™.

**Methods:**

From 2017 to 2020, we prospectively followed 91 consecutive patients who underwent a Bentall procedure with a BioConduit™ valved graft in our institution. The primary outcomes were aortic bioprosthetic valve dysfunction and mortality according to Valve Academic Research Consortium 3 (VARC3).

**Results:**

Mean age was 70 ± 10 years and 67 patients (74%) were men. Ascending aortic aneurysm (72%), aortic valve regurgitation (51%) or stenosis (20%) and acute endocarditis (14%) were the main indications for surgery. Seventy-four patients (81.3%) were followed up at 1 year. The perioperative mortality was 8% (n = 8), the early, 1 year, mortality was 2% (n = 2) and the midterm mortality, at 4 years of follow up, was 4% (n = 3). Ten patients fulfilled the criteria for hemodynamic valve deterioration at 1 year (13%) and 14 for a bioprosthetic valve failure during the entire follow-up (17%).

**Conclusions:**

We are reporting early and midterm results of Bentall procedures with the all-biological, valved, No-React® BioConduit™. To our knowledge, this is the first study reporting an early and midterm unexpectedly high rate of non-structural prosthetic hemodynamic deterioration. The rate of endocarditis and atrioventricular disconnections remain similar to previous studies.

**Supplementary Information:**

The online version contains supplementary material available at 10.1186/s13019-022-02073-5.

## Background

The Bentall procedure is the gold standard therapy in patients with either ascending aorta or aortic root aneurysm combined with aortic valve disease precluding a valve sparing procedure. [1]. The original technique described by Bentall and De Bono using a composite mechanical valved graft benefited from iterative refinements in order to overcome specific surgical drawbacks [2]. Nowadays, either preassembled or self-assembled conduits, associating tubular straight or Valsalva graft and biological or mechanical valve, are widely used [3, 4]. As an alternative, a fully xenobiological stentless valved conduit, the Shelhigh NR-2000, was introduced in the late 1990’s and thereafter withdrawn from the market. Recently, a totally biological stentless conduits have been reintroduced in a modified version, using a porcine aortic valve and a bovine pericardial tube (BioIntegral™, BioValsalva™). The goal of our study was to investigate the early and midterm results of the Bentall procedure using BioIntegral™ BioConduit™ in our single-center experience.

## Methods

### Study design

It was a prospective observational study, without control-group, carried out from 2017 to 2020 in our tertiary centre.

### Study population

All consecutive patients undergoing a Bentall procedure with the BioIntegral™ Surgical BioConduit™ No-React® in our institution (n = 91) were prospectively included during the study period. All patient data were collected from hospitals’ medical records. Cohort patients underwent an aortic root replacement with an all-biological graft in cases of complex endocarditis or redo surgeries, cases of patients who were not candidates for an autograft procedure but a mechanical graft was not indicated and in elderly patients with degenerative aortic root disease.

The study is conformed to the principles outlined in the Declaration of Helsinki. According to French law on ethics, patients were informed that their codified data would be used for the study. The patient also provided informed written consent for the publication of the study data. The Institutional Review Board of the Rangueil University Hospital of Toulouse, France, approved the study protocol and the publication of data (number RnlPH 2022-50) on the 7th of April 2022.

### Surgical data

All interventions were performed by four senior surgeons. All patients were operated through median sternotomy. Standard cardiopulmonary bypass, aortic cross-clamp and anterograde and retrograde cold blood potassium-enriched cardioplegia were used in all patients. When an aortic arch replacement was performed, moderate hypothermic circulatory arrest and selective antegrade cerebral perfusion through the right axillary artery were associated. After aortic cross-clamping, the aortic valve, the aortic root and the ascending aorta were excised, followed by aortic annulus decalcification when required. The coronary ostia were isolated with their buttons. After sizing of the aortic annulus, the prosthesis was chosen, with a trend in oversizing. The all-biological stentless valved bioconduit is designed to offer similar hemodynamics as the native aortic valve and theoretically aimed to insure a larger post-operative effective orifice area (EOA). The conduits’ No-React® treatment aims to reduce the grafts’ related infections and calcifications as well as to prevent remodeling and graft aneurysmal dilatation [5–7]. As the traditional glutaraldehyde preserved biological valves tend to calcify, the No-React® detoxification process promises to eliminate residual glutaraldehyde and to ensure stable tissue cross-linking, resulting in less or no calcification or tissue deterioration in the animal model [5, 6]. A variety of BioConduit™ sizes between 21 to 29 mm were implanted. The trend of oversizing refers to choosing a conduit one-size oversized following the manufacturer’s recommendations, to achieve greater effective surface areas, avoiding mismatch [7]. Depending on operator preferences, two types of implantation techniques were used, either multiple single, interrupted, non-everting, reinforced U-stiches to implant the composite graft in supra-annular position (n = 33, 36%) or everting stitches to implant the prosthesis in intra-annular position (n = 58, 64%). Then, 2 holes were made in the tubular graft and both coronary ostia were reimplanted by running sutures (Prolene 6–0). Fibrin glue was used in most cases to reinforce sutures.

### Imaging protocol

A post-operative transthoracic echocardiographic (TTE) assessment was performed before hospital discharge: 2D TTE standard views were obtained using a standard ultrasound system using a 1–5 MHz probe (VIVID S70, GE Healthcare). A new TTE assessment was conducted at 1 year, using the same system. EOA was calculated by the continuity equation method. Aortic annulus diameter was measured at mid-systole, from the parasternal long-axis view, at the level of the prosthetic annulus, in a zoomed mode, from inner-edge to inner-edge. The velocity–time integral of blood flow was measured in the left ventricular outflow track by pulsed doppler. Mean transaortic gradient and maximal velocity were evaluated by transprosthetic continuous wave doppler. The doppler velocity index was calculated as the ratio of the proximal peak flow velocity in the LVOT to the transprothetic peak flow velocity. All examinations were interpreted blindly on a dedicated workstation (EchoPac 204 GE Healthcare) by two operators. In addition, during the follow-up, in case of bioprosthetic valve failure (BVF), a TEE and a cardiac CT were performed.

### Study outcomes

The endpoints used were those proposed by the Valve Academic Research Consortium 3 (VARC3).

#### Mortality [8]

- Periprocedural mortality was defined as all-cause mortality occurring ≤ 30 days after the index procedure or occurring > 30 days but during the index hospitalization.
- Early mortality was defined as death occurring > 30 days but ≤ 1 year after the index hospitalization.
- Midterm mortality was defined as death occurring > 1 year after the index hospitalization but ≤ 4 years, at end of follow up.

#### Aortic bioprosthetic valve dysfunction (BVD)

- In terms of etiology, BVDs were defined as structural valve deterioration (SVD), nonstructural valve dysfunction (NSVD), thrombosis or endocarditis. SVD was reported as intrinsic permanent changes of the prosthetic valve [9]. NSVD was reported as any abnormality not intrinsic to the bioprosthesis, resulting in its malfunction (e.g. pannus, prosthesis-patient mismatch) [9].
- Hemodynamic valve performance assessment was protocolized at 1 year of the index procedure. Moderate hemodynamic valvular deterioration (HVD) was defined as an increase in the transaortic mean gradient of $\ge$ 10 mmHg resulting in a mean gradient of $\ge$ 20 mmHg, with concomitant decrease in the EOA $\ge$ 0.3 cm^2^ and decrease in doppler velocity index $\ge$ 0.1 compared to the post-operative assessment. Severe HVD was defined as an increase in the mean gradient of $\ge$ 20 mmHg resulting in a mean gradient of $\ge$ 30 mmHg, with concomitant decrease in the EOA $\ge$ 0.6 cm^2^ and decrease in doppler velocity index $\ge$ 0.2 [9].
- During the follow-up, the occurrence of a BVF was considered as an endpoint. Finally, BVF was defined by the occurrence of BVD associated with clinically expressive criteria (heart failure symptoms, fever, angina, ischemic event), irreversible severe HVD, aortic valve reoperation or re-intervention or valve-related death [9].

### Statistical analysis

Continuous variables were expressed as means ± standard deviation or as medians with interquartile ranges (IQR) when not normally distributed. Nominal variables were expressed as numbers and percentages. The association between the mean values of continuous variables was assessed using the Mann–Whitney rank sum test. Nominal variables were investigated by the χ^2^ test or the Fisher exact test when appropriate. The software XLSTATS v2019.1 (Addinsoft, Paris, FR) was used for statistical analysis.

## Results

### Population

This study included 91 patients, mostly men (74%) with a mean age at intervention of 70 ± 10 years. Preoperative patient’s characteristics are reported in Table 1. Most patients had an ascending aortic aneurysm (n = 65, 72%). There were 22 cases of redo procedures (24%). Sixteen procedures were performed on an emergency basis (18%), including 10 cases of type A acute aortic dissection (11%), 12 cases of severe prosthetic endocarditis (13%) and 1 complex of native aorto-mitral endocarditis. Forty-four patients underwent combined interventions (48%): coronary artery bypass graft in 22 cases (24%), mitral valve repair or replacement in 5 (6%) and aortic arch or hemiarch replacement in 14 (15.3%) cases.

**Table 1:** Patients and intra-operative characteristics (n = 91)

Age (years) Mean (SD)	70 ± 10
Gender	
Male	67 (74%)
NYHA Class at operation	2 ± 1
Comorbidities	
Renal insufficiency (GFR < 60 ml/min)	17 (17%)
Marfan syndrome	1 (1%)
Indication	
Ascending aortic aneurysm	65 (72%)
Type A aortic dissection	10 (11%)
Acute endocarditis on native valve	1 (1%)
Acute endocarditis on prosthesis	12 (13%)
Aortic valve regurgitation	46 (51%)
Aortic stenosis	18 (20%)
Mixed aortic valve lesion	
Bicuspid aortic valve	28 (31%)
Emergency	16 (18%)
Redo Surgery	22 (24%)
CPB time (min, mean)	114 ± 52
Aortic cross clamp time (min, mean)	93 ± 46
Extension of the aorta replacement	
Root and ascending aorta	76 (84%)
Root, ascending aorta and partial arch	9 (10%)
Root and ascending aorta and total arch	5 (5%)
Total Elephant Trunk	1 (1%)
Concomitant procedure	
CABG	22 (24%)
Mitral valve procedure (repair/replacement)	5 (6%)
Tricuspid annuloplasty	3 (3%)
AF ablation and Left Atrial Appendage Closure	5 (5%)
Combination	44 (48%)
Size of graft implanted	
21 mm	1 (1%)
23 mm	12 (13%)
25 mm	26 (29%)
27 mm	39 (43%)
29 mm	13 (14%)
Surgical technique	
Interrupted single, non-everting, pledged, reinforced U-stiches (supra-annular level)	33(36%)
Interrupted single, everting, pledged, reinforced U-stiches (intra-annular level)	58(64%)

AF—atrial fibrillation, CABG—coronary artery bypass graft; CPB—cardiopulmonary by-pass, GFR—Glomerular filtration rate, NYHA—New York Heart Association

Among the 83 patients discharged from hospital, 9 were lost to follow-up (10%). Median follow-up was 4 years.

### Mortality

The periprocedural mortality rate was 8% (n = 8). Among them, 3 patients with a pre-operative severe left ventricular dysfunction died shortly after the procedure from low cardiac output and multiorgan failure syndrome, 2 patients operated for complicated infective endocarditis on previous Bentall prosthesis died in the first 24 h from refractory septic vasoplegic syndrome, 2 patients operated for acute type A aortic dissection died either from massive hemorrhagic stroke or acute right ventricular failure in the first week after surgery and 1 patient experienced acute respiratory distress syndrome.

Early mortality rate was 2%. Among the 83 patients discharged from hospital, 2 died in the first year, both of them experiencing graft proximal anastomosis partial detachment at 9 and 10 months, respectively. Both were reoperated, but died from hemorrhagic stroke or massive intraoperative bleeding, respectively.

Midterm mortality rate was 4% (3 patients). One 62 years-old male, who had already undergone two Bentall procedures presented a recurrent bacterial graft endocarditis at 18 months after discharge. He was referred to surgery and died in the operating room from uncontrolled bleeding. One 83 years-old patient died from respiratory distress related to a severe form of Covid-19 pneumonia. The third, 72 years-old male died from metastatic pulmonary adenocarcinoma.

### Bioprosthetic valve dysfunction

#### Early results

Regarding the prosthetic hemodynamic features, despite normal postoperative hemodynamic profiles, without signs of obstruction, we observed a decrease in graft performance at 1 year, mainly in terms of EOA (1.3 ± 0.2 cm^2^ vs 0.9 ± 0.4 cm^2^, p = 0.02 for 23 mm graft, 1.6 ± 0.4 cm^2^ vs 1.2 ± 0.5 cm^2^, p = 0.01 for 25 mm graft, 1.9 ± 0.5 cm^2^ vs 1.5 ± 0.5 cm^2^, p < 0.01 for 27 mm graft, 2.1 ± 0.6 cm^2^ vs 1.6 ± 0.6 cm^2^, p = 0.17 for 29 mm graft) but also in terms of transaortic mean gradient and maximum velocity. [10, 11].

At the 1-year follow-up, 10 patients fulfilled criteria for HVD (13%), 6 being moderate and 4 severe. The factors associated with HVD are presented in Table 2. Preoperative characteristics were not associated with HVD. The implanted graft caliber was significantly associated with the occurrence of HVD at 1 year (8 (80%) vs. 21 (33%), *p* = 0.01 for 23- or 25-mm graft), especially in the smallest sizes.

**Table 2:** Factors associated with the presence of hemodynamic valve deterioration (HVD) at 1 year (n = 74)

	No HVD at 1 year n = 64	Moderate or severe HVD at 1 year n = 10	*p* Value
Characteristics			
Age (years)	71 ± 11	72 ± 10	0.75
Female gender	14 (22%)	5 (50%)	0.11
AHT	41 (64%)	9 (90%)	0.15
Diabetes	11 (17%)	2 (20%)	1
Renal insufficiency (GFR < 60 ml/min)	**8 (12%)**	**4 (40%)**	**0.05**
Marfan’s syndrome	1(2%)	0 (0%)	1
Preoperative LVEF (%)	56 ± 10	51 ± 7	0.07
Indication			
Ascending aortic aneurysm	48 (75%)	9 (90%)	0.43
Type A aortic dissection	5 (8%)	1 (10%)	1
Acute endocarditis	11(17%)	0 (0%)	0.34
Aortic valve regurgitation	29 (45%)	7 (70%)	0.18
Aortic stenosis	13 (20%)	4 (40%)	0.22
Bicuspid aortic valve	21 (33%)	4 (40%)	0.72
Surgical data			
Emergency	10 (16%)	1 (10%)	1
Redo Surgery	17 (26%)	0 (0%)	0.10
CPB time (min, mean)	112 ± 50	103 ± 40	0.55
Aortic cross clamp time (min, mean)	87 ± 40	81 ± 32	0.76
CABG	14 (22%)	2 (20%)	1
Mitral valve procedure (repair/replacement)	3 (5%)	0 (0%)	1
Tricuspid annuloplasty	2 (3%)	0 (0%)	1
AF ablation and Left Atrial Appendage Closure	3 (5%)	1 (10%)	0.44
Combination	28 (44%)	3 (30%)	0.50
Size of graft implanted	**26 ± 2**	**25 ± 2**	** < 0.01**
23/ 25 mm graft	**21 (33%)**	**8 (80%)**	**0.01**
Interrupted single, everting, pledged, reinforced U-stiches (intra-annular level) (vs Interrupted single, non-everting, pledged, reinforced U-stiches (supra-annular level)	40 (62%)	8 (80%)	0.47
Echocardiographic data			
Post-operative Vmax (m/s)	2.2 ± 0.5	2.5 ± 0.5	0.06
Post-operative Mean gradient (mmHg)	12 ± 6	15 ± 6	0.11
Post-operative aortic valve effective surface area (cm^2^)	1.8 ± 0.5	1.8 ± 0.6	0.99
Post-operative indexed aortic effective surface area (cm^2^/m^2)^	1 ± 0.2	0.9 ± 0.3	0.91
1 Year Vmax (m/s)	**2.3 ± 0.4**	**3.7 ± 0.7**	** < 0.01**
1 Year Mean gradient (mmHg)	**12 ± 5**	**35 ± 14**	** < 0.01**
1 Year aortic valve effective surface area (cm^2)^	**1.5 ± 0.5**	**0.8 ± 0.3**	** < 0.01**
1 Year aortic indexed effective surface area (cm^2^/m^2)^	**0.8 ± 0.3**	**0.5 ± 0.1**	** < 0.01**
1 Year LVEF (%)	58 ± 11	59 ± 6	1

AF—atrial fibrillation, AHT—arterial hypertension, CABG—coronary artery bypass graft; CPB—cardiopulmonary by-pass, GFR—Glomerular filtration rate, HVD—hemodynamic valve deterioration; LVEF—left ventricular ejection fraction; Vmax—maximum velocity; Continuous variables were expressed as means ± standard deviation. Nominal variables were expressed as numbers and percentages in bold: *p* value ≤ 0.05

However, the patients who secondarily developed HVD had similar hemodynamic parameters as the rest of the cohort at the pre-discharge postoperative exam.

There was 1 case (1.3%) of early graft endocarditis with negative blood cultures, diagnosed at 8 months after surgery. We treated it medically.

We noted three cases (4.1%) of partial proximal anastomosis disruption: two cases with total atrio-ventricular disruption with large false aneurysms at the level of the proximal anastomosis at 9 months and respectively at 10 months after the initial surgery. We did not find any argument in favor of an infective endocarditis. Moreover, one patient’s initial surgery was in an elective setting, for a degenerative aortic aneurysm. The third patient had a similar aortic root disruption, but it appeared 4 months after a Bentall intervention for a complex aortic and mitral endocarditis with fragile tissues.

### Midterm results

Five patients experienced infective endocarditis (6.8%) at a median of 1 year and 4 months. One patient was treated surgically and the other 5, medically.

There weren’t observed any more cases of hemodynamic dysfunction or AV disruptions until follow up was closed.

Regarding early and midterm results, we observed a total of 14 patients presenting a BVF during follow-up (17%). A re-intervention was performed in 8 cases (10%). Three patients had a valve-in-valve TAVR for severe HVD. Five patients had open redo surgery: three cases of partial proximal anastomosis detachment, one case of graft endocarditis with valvular involvement and one severe HVD. Among them, 3 patients died related to the procedure.

Regarding etiologies of BVF, 6 patients experienced infective endocarditis (8%), 3 patients had partial proximal anastomosis detachment (4%) and the other 4 patients had severe irreversible HVD (5.5%). All patients with endocarditis underwent TTE and TEE which were abnormal in two cases, highlighting valve involvement (aortic vegetations (n = 2), aortomitral abscess (n = 1) and pseudoaneurysm (n = 1)). Endocarditis patients also underwent a CT scan which showed in all cases a proximal collection around the biological graft with peripheral contrast diffusion, without signs of a pseudoaneurysm. PET combined with CT (PET/CT) was also performed in four cases, showing an abnormal intense uptake on the graft collection and/or the valvular prosthesis. Regarding the pathogens involved, there were 2 cases of staphylococcus involvement (aureus and epidermidis), 1 with Enterobacter Aerogenes, 1 with E. coli, another case a streptococcus oralis infection and lastly, 1 with negative blood cultures.

**Fig. 1:**
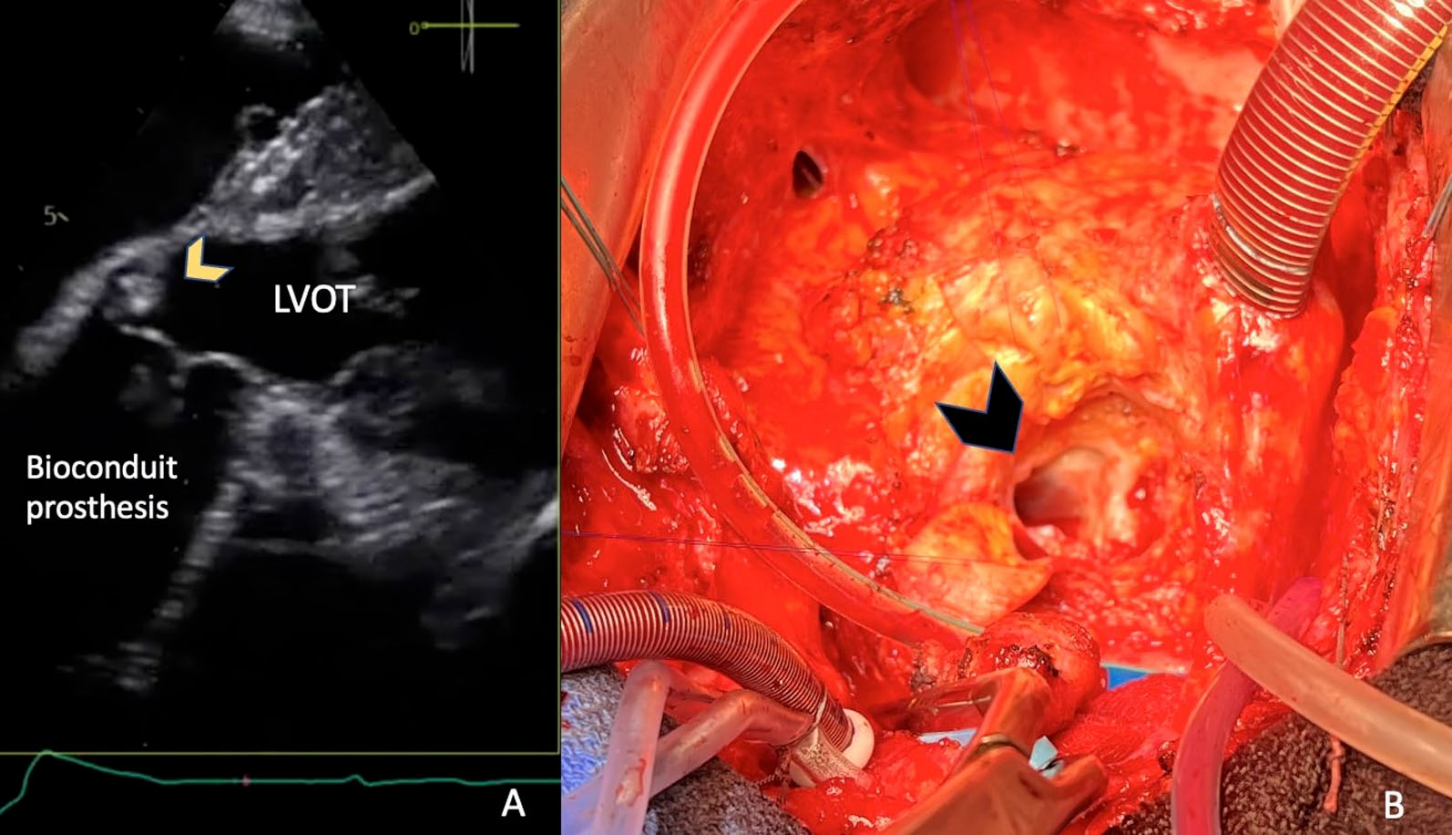
**A** Transesophageal echocardiography, deep transgastric view, annular hyperechogenic structure (yellow arrow); LVOT—left ventricle outflow tract; **B** Intraoperative view with the annular diaphragmatic structure (black arrow) visualized after complete prosthetic dissection

For all patients with HVD, thrombosis was ruled out by CT, and endocarditis by Duke criteria. SVD was eliminated by TEE and CT. Of note, none of these patients had regurgitation. Regarding NSVD, a patients-prosthesis mismatch did not appear to be involved, as postoperative indexed EOA was not associated with the occurrence of HVD at 1 year. Finally, for all patients with HVD, imaging found a 3–4 mm, circumferential, hyperechogenic ring, located at the level of the prosthetic aortic annulus and at the graft’s proximal anastomosis towards its’ ventricular side, near the pledgets. In these patients, on CT and TEE, there was no structural prosthetic abnormality (neither fibrosis, calcification, leaflet tear and wear, hypo-attenuated leaflet thickening nor thrombosis). Moreover, we have randomly reviewed patients’ echocardiographic exams concluding that the circumferential structure is a common finding in patients being implanted with this type of biological graft. Indeed, 29 patients had a reduction of their aortic annulus size at 1 year (39%). We identified this annular structure in the per-operatory setting, during the reintervention for a failing bioprosthesis (Fig. 1).

## Discussion

This prospective study reports the midterm outcomes of 91 consecutive patients who underwent a Bentall procedure with the BioConduit™ No React® between 2017 and 2020, in our institution. Main results are as follows: 1) the hemodynamic performances at 1 year were unsatisfying, with an overall trend towards reduction in EOA and a significant rate of HVD, 2) despite the No-react® treatment of the conduit, graft endocarditis was not rare and 3) we observed some cases of early graft detachment at its proximal anastomosis with the aortic root.

Biological bioconduits, by avoiding stent and sewing cuff at the annular level, are intended to achieve more physiological flow pattern and thus superior hemodynamics [7, 12, 13]. However, our study identified a high rate of HVD and a general decrease in EOA at 1 year as being related to prosthesis of smaller calibers (23 and 25 mm) (*p* < 0.001) and renal insufficiency (*p* < 0.001) (Table 2). Even though we observed a general reduction in the annulus diameter, we think it had a more rapid obstructive impact in initially smaller calibers.

**Fig. 2:**
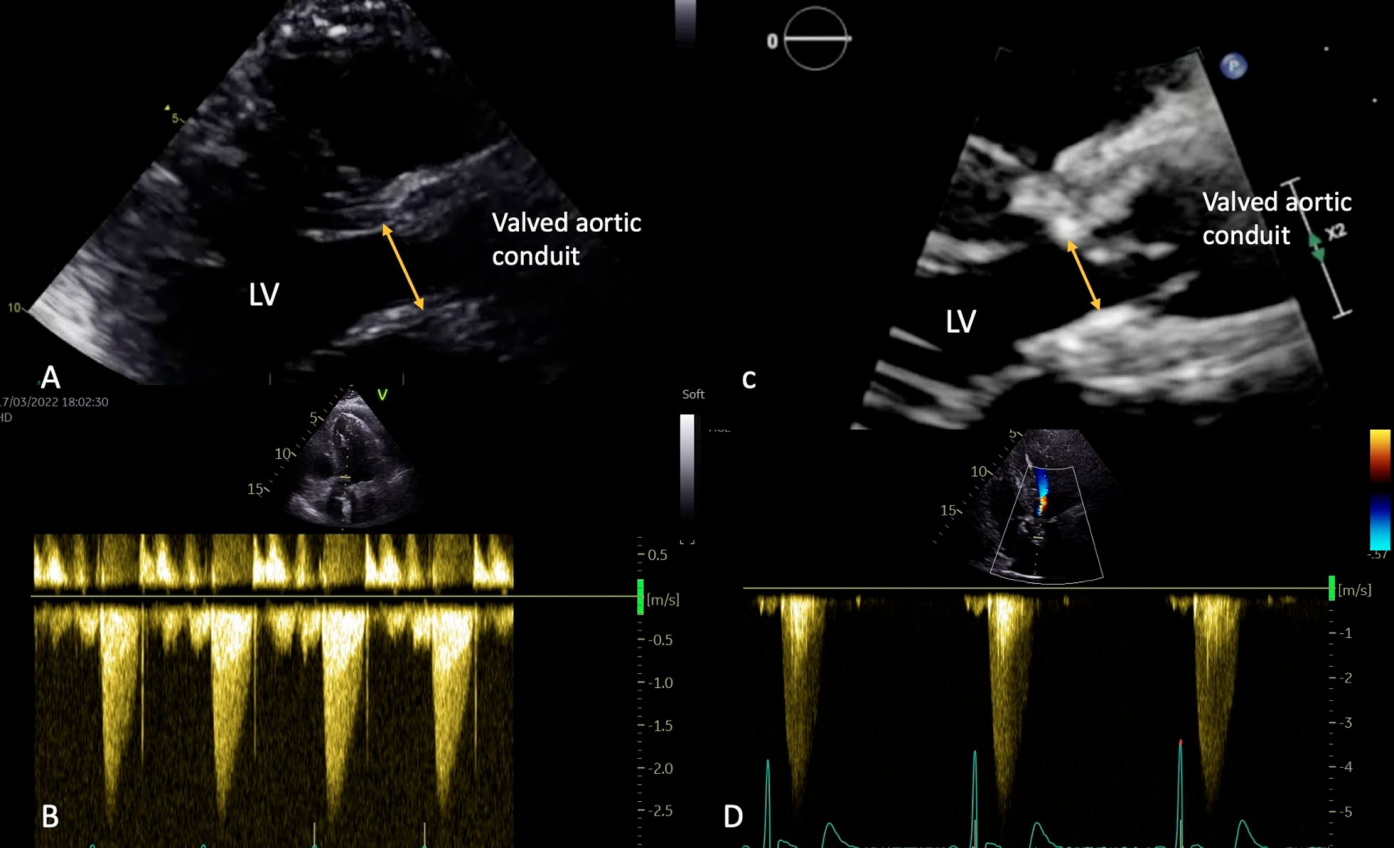
Transthoracic echocardiography, long axis parasternal view with same patient early post-operatory (**A**) and (**C**) and 1-year follow up exam (**B**) and (**D**), showing a shrinkage of the aortic annulus at 1 year (measured at the level of the yellow arrows), from 18 to 14 mm (**A**) and (**C**) together with a transaortic mean gradient and maximal velocity by Doppler continuous wave interrogation, from 2,4 m/sec and 12 mmHg (**B**) to 4,6 m/sec and 59 mmHg (**D**)

Regarding patients with HVD, our imaging protocol allowed us to rule out endocarditis, thrombosis or SVD in all cases. Our main observation explaining this phenomenon causing NSVD might be similar to a pannus formation. Firstly, we noticed a reduction in the aortic annulus diameter at 1 year (Fig. 2). In addition, we found a 3–4 mm, external circumferential, hyperechogenic, non-perfused ring, situated at the level of the prosthetic aortic annulus and at the graft’s proximal anastomosis near the pledgets. This structure was identified during our imaging protocol and confirmed in the perioperative setting (Fig. 1). We concluded that HVD was probably related to the external circumferential diaphragm that explained the shrinkage of the aortic diameter. Nevertheless, it is interesting to notice the rapid development of this “pannus-like” structure, in less than a year (Additional files 1, 2, 3). Concerning its potential cause, several hypotheses are put forward. It may be related to the surgical techniques as the structure was near the pledgets used for our surgical interrupted suture, reinforced with biological fibrin glue.

As for the surgical implantation technique, we used either a multiple, single, interrupted, non-everting, pledged reinforced U-stiches to implant the composite graft in supra-annular position (n = 33, 36%) or everting stitches to implant the prosthesis in intra-annular position (n = 58, 64%). After univariate analysis, we found no correlation between the technique and the event of bioprosthetic valve dysfunction (Table 2, *p* = 0.47).

We can also consider the role of the immune system. The glutaraldehyde bioconduit treatment is meant to eliminate immunogenic proteins [14]. Nevertheless, multiple observational studies identified xenograft porcine components (extracellular matrix specific glycans, galactose-a-1,3-galactose and N-glycolylneuraminic acid) who trigger host antibody formation [15–19]. Sustaining the same immunogenic hypothesis, a group from Munich studied the effects of a pericardial porcine, No-React® patch and they found sterile abscess formation that was suspected to be an immunogenic reaction, a xenogeneic complement-mediated graft rejection [20]. The question of patient-prosthesis mismatch can be raised because the smallest sizes of prostheses seemed to be more sensitive to hemodynamic deterioration. We believe that this observation is not related to an initial mismatch but to the fact that a decrease in EOA logically has more impact on a small prosthesis than on a larger one.

In light of all of the above, we speculate an early inflammatory reaction could have been triggered by the biological conduit itself, favored by the use of biological glue or/and by the surgical technique (all patients were implanted using a multiple, single, interrupted, everting or non-everting suture with pledged reinforced U-stiches). Lastly, concerning the surgical technique, we note that other teams (Carrel et al., Stefanelli et al., Sahin et al., Kaya et al., Galinanes et al.) reported using as technique of conduit implantation either a running suture or an interrupted, nevertheless, they were not confronted with this problem [13, 21–24].

Six patients were diagnosed with graft endocarditis. Diagnosis was made according to the modified Duke criteria and was often difficult, due to a combination of atypical clinical, biological, radiological and echocardiographic observations [25]. These features are not specific to this conduit and are usual after Bentall surgery. These results are surprising insofar as one of the advantages put forward in favor of these conduits is their expected low rate of reinfection.

In this regard, Galinanes et al. reported excellent late results (1 case of conduit infective endocarditis in 10 years) [13]. Siniawski et al., Musci et al. and Wendt et al. suggested the same reassuring results [26, 27, 31]. Recently, Stefanelli et al. also reported satisfactory results, with freedom from the BioConduit® graft infection of 95.7% at 5 and 15 years (CI 0.95) [22]. However, larger cohorts are still needed to confirm these results.

Previous studies reported a high number of aorto-ventricular disconnections especially related to the initial Shelhigh’s biological conduit [21, 23, 24, 28]. The Shelhigh graft was a stentless, valved, No-React® biological conduit implanted mostly in the early 2000s. Due to unexpected conduit disconnections, in 2007, the United States FDA published a notification, the product being retried of the market [28, 30]. Despite this drawback, in 2013, Musci et al. published reassuring results after 11 years of follow-up of 255 patients implanted with Shelhigh initial graft [30]. The Berlin group reported a low reinfection rate (0.78% early and 2.35% late reinfection) concluding that patients’ outcome was dependent of their surgical urgency. Afterwards, a new version of the graft, the BioIntegral Surgical, BioConduit™ was released on the market. Regarding its predecessor (the Shelhigh conduit), while some teams report relatively satisfactory early and late follow-up results (Galinanes et al., Musci et al.), other teams report some dreadful complications (Sahin et al., Kaya et al., Carrel et al., Reineke et al., Sadeque et al.) referring to the high rate of endocarditis and of atrio-ventricular disconnections with proximal false aneurysm formation [13, 21, 23, 24, 27–30, 32]. In contrast, they report conduit hemodynamic dysfunction only in a few cases. Reineke et al. describes 8 cases (2.3%) with structural valve deterioration at late follow up. In contrast, Kaya et al. reports 3 cases (1.7%) with hemodynamic failure at early follow-up, without detailing. Concerning the BioConduit™, Stefanelli et at publishes satisfactory early and long-term results [22]. In our series, we report 10 cases (13%) presenting criteria for non-structural hemodynamic valve dysfunction at 1 year and 4 of them fulfilling criteria for failure, needing reintervention. This was an unexpected finding, as to our knowledge, such a high incidence of biological conduit hemodynamic dysfunction was never cited before.

There are several limitations of this study. This was a prospective but single-center, non-randomized study with lack of group control. Even though there were 91 patients included, the population analyzed was rather small and heterogenous, gathering elective as well as emergent cases. Being a single institution study, with 4 surgeons and 2 techniques, while representing a limitation, could contribute to reducing biases related to the use of multiple techniques.

## Conclusions

This study reports the early and midterm results of the newest all-biological valved conduit. Even though it is designed to achieve superior hemodynamics by excluding the valvular stent, our study reveals an abnormally high rate of early prosthetic non-structural dysfunction and failure that appears to be mostly related to a multifactorial progressive shrinkage of the aortic annulus. Regarding graft infections, we observed that endocarditis is not rare, despite conduits all-biological structure. Lastly, there are still some cases of conduit proximal anastomotic detachments.

## Supplementary Information

**Additional file 1.** Video 1 Transthoracic echocardiography, long axis parasternal view. Exam at 1-year follow up exam showing a diminished and hyperechogenic, with a pannus-like structure at the level of the aortic annulus.

**Additional file 2.** Video 2 Transthoracic echocardiography, long axis parasternal view. Exam at 1-year follow up exam showing a diminished and hyperechogenic, with a pannus-like structure at the level of the aortic annulus.

**Additional file 3.** Video 3 Transthoracic echocardiography, long axis parasternal view. Exam at 1-year follow up exam showing a diminished and hyperechogenic, with a pannus-like structure at the level of the aortic annulus.

## Data Availability

The datasets and the materials used and analyzed during the current study are available from the corresponding author on reasonable request.

## Abbreviations

BVD: Bioprosthetic valve dysfunction
BVF: Bioprosthetic valve failure
EOA: Effective orifice area
HVD: Hemodynamic valve deterioration
NSVD: Nonstructural valve deterioration
PPM: Patient-prosthesis mismatch
SVD: Structural valve deterioration
TAVR: Transcatheter aortic valve replacement
TEE: Transesophageal echocardiogram
TTE: Transthoracic echocardiogram
